# Diagnostic accuracy of SARS-CoV-2 rapid antigen test from self-collected anterior nasal swabs in children compared to rapid antigen test and RT-PCR from nasopharyngeal swabs collected by healthcare workers: A multicentric prospective study

**DOI:** 10.3389/fped.2022.980549

**Published:** 2022-09-21

**Authors:** Robert Cohen, Camille Aupiais, Anne Filleron, Fabienne Cahn-Sellem, Olivier Romain, Stéphane Béchet, Anne Auvrignon, Christophe Batard, Brigitte Virey, Camille Jung, Alexis Rybak, Corinne Levy

**Affiliations:** ^1^Association Clinique et Thérapeutique Infantile du Val-de-Marne (ACTIV), Créteil, France; ^2^Clinical Research Center, Centre Hospitalier Intercommunal de Créteil, Créteil, France; ^3^Université Paris Est, IMRB-GRC GEMINI, Créteil, France; ^4^Association Française de Pédiatrie Ambulatoire (AFPA), Orléans, France; ^5^Groupe de Pathologie Infectieuse Pédiatrique (GPIP), Créteil, France; ^6^Assistance Publique–Hôpitaux de Paris, Clinical Epidemiology Unit, Robert Debré University Hospital, ECEVE INSERM UMR 1123, Université de Paris, Paris, France; ^7^Assistance Publique–Hôpitaux de Paris, Pediatric Department, Jean Verdier University Hospital, Université Sorbonne Paris Nord, Bondy, France; ^8^Department of Pediatrics, CHU Nîmes, University of Montpellier, Nîmes, France; ^9^Assistance Publique–Hôpitaux de Paris, Neonatalogy Department, Antoine Béclère University Hospital, Université Paris Saclay, Clamart, France; ^10^Assistance Publique–Hôpitaux de Paris, Pediatric Emergency Department, Robert Debré University Hospital, Université de Paris, Paris, France

**Keywords:** COVID-19, ambulatory setting, test, pediatric, self-test

## Abstract

Testing for SARS-CoV-2 is central to COVID-19 management. Rapid antigen test from self-collected anterior nasal swabs (SCANS-RAT) are often used in children but their performance have not been assessed in real-life. We aimed to compare this testing method to the two methods usually used: reverse transcription polymerase chain reaction from nasopharyngeal swabs collected by healthcare workers (HCW-PCR) and rapid antigen test from nasopharyngeal swabs collected by healthcare workers (HCW-RAT), estimating the accuracy and acceptance, in a pediatric real-life study. From September 2021 to January 2022, we performed a manufacturer-independent cross-sectional, prospective, multicenter study involving 74 pediatric ambulatory centers and 5 emergency units throughout France. Children ≥6 months to 15 years old with suggestive symptoms of COVID-19 or children in contact with a COVID-19–positive patient were prospectively enrolled. We included 836 children (median 4 years), 774 (92.6%) were symptomatic. The comparators were HCW-PCR for 267 children, and HCW-RAT for 593 children. The sensitivity of the SCANS-RAT test compared to HCW-RAT was 91.3% (95%CI 82.8; 96.4). Sensitivity was 70.4% (95%CI 59.2; 80.0) compared to all HCW-PCR and 84.6% (95%CI 71.9; 93.1) when considering cycle threshold <33. The specificity was always >97%. Among children aged ≥6 years, 90.9% of SCANS-RAT were self-collected without adult intervention. On appreciation rating (from 1, very pleasant, to 10, very unpleasant), 77.9% of children chose a score ≤3. SCANS-RAT have good sensitivity and specificity and are well accepted by children. A repeated screening strategy using these tests can play a major role in controlling the pandemic.

## Introduction

Following the successive COVID-19 waves due to several SARS-CoV-2 variants, in many countries, healthcare authorities implemented non-pharmaceutical interventions and large-scale testing strategies (1, 2). Two methods were mainly used without distinction in France: reverse transcription polymerase chain reaction from nasopharyngeal swabs collected by healthcare workers (HCW-PCR), and rapid antigen test from nasopharyngeal swabs collected by healthcare workers (HCW-RAT) in addition to immunization programs (3). In 2021, 48.8% of the 168 million of tests recorded in the French national database were HCW-RAT (4). While HCW-RAT has lower analytical sensitivity than HCW-PCR, this method is highly specific, inexpensive, and provides results in minutes.

Testing for SARS-CoV-2 is central to COVID-19 management and essential to detect people who are likely infectious, helping to implement control measures (5). For SARS-CoV-2 testing and screening, especially in children, rapid antigen test from self-collected anterior nasal swabs (SCANS-RAT) could be a useful tool (5). The duration of the pandemic and the frequency with which testing must be done to limit infectiousness, particularly in schools, means that the less invasive, less painful and less unpleasant tests should be used, to avoid poor acceptance by children and families.

The diagnostic accuracy of HCW-RAT for diagnosing SARS-CoV-2 infection in children has been assessed in several studies and was the subject of a meta-analysis (6). No test included fully satisfied the performance requirements recommended by the World Health Organization, and the diagnostic accuracy of the HCW-RAT under real-life conditions varied broadly (6). A recent French study in an emergency department found good sensitivity of the HCW-RAT in real life for symptomatic children, and when focused on high viral load, the sensitivity was excellent (7). Nasopharyngeal swabbing compared to other upper-respiratory sampling methods, including oropharyngeal swab, appeared to be superior in a pediatric study finding a significantly higher positivity rate and a significantly higher viral load on nasal samples (8). In adults, the diagnostic accuracy of SCANS-RAT was assessed in several studies, but relatively few patients were enrolled (9–12). Millions of SCANS-RAT are used each day worldwide, but to our knowledge, no study has assessed their performance in real-life in children.

This study compared SCANS-RAT to HCW-PCR and HCW-RAT, estimating the accuracy and acceptance, in a pediatric real-life study.

## Methods

From September 10, 2021, to January 29, 2022, the Association Clinique et Thérapeutique Infantile du Val de Marne (ACTIV) network conducted a manufacturer-independent cross-sectional, prospective, multicenter study involving 74 pediatric ambulatory centers (see the Acknowledgments section) and 5 emergency units (Jean-Verdier hospital in Bondy, intercommunal hospital of Créteil, Princess Grace hospital in Monaco, Carémeau hospital in Nîmes, and Versailles hospital in Le Chesnay) throughout France. Children ≥ 6 months to 15 years old with suggestive symptoms of COVID-19 or children in contact with a COVID-19–positive patient were prospectively enrolled.

Healthcare workers collected nasopharyngeal swabs to perform a rapid antigen test during the medical visit. Reverse transcription polymerase chain reaction were collected either during the medical visit or with a medical prescription in a laboratory. Swabs were performed as recommended in international guidelines (13). Ambulatory and hospital virology laboratories analyzed the HCW-PCR specimens according to the French National Reference Center recommendations (14). At the same time, SCANS-RAT was offered to children in the pediatrician office or emergency department. After oral instructions from an adult (parents or pediatricians), children self-collected the nasal specimen from both nares. Adults could help to perform the test when the children were not able to perform the swabbing alone. The test used was COVID-VIRO ALL IN® (AAZ-LMB, Boulogne Billancourt, France) which has a short soft sponge sampling part (1.5 cm). Recommended sampling duration was 30 s (15 s per nostril) (15). Children were asked to rate the SCANS-RAT from 1, very pleasant, to 10, very unpleasant.

After informing the parents of the participating children about the study, an electronic case report form in a secure database was prospectively completed by the pediatrician. Any child or parent had the right to object to the data collection for this study.

The diagnosis accuracy of the SCANS-RAT was compared with that of HCW-PCR and/or HCW-RAT. According to the spread of different variants in France, we defined 2 periods: period 1, when the Delta variant was predominant and Omicron not yet or poorly isolated in France (from September 10, 2021 to December 19, 2021), and period 2, when the Omicron variant was spreading and became predominant (i.e., > 50%, from December, 20, 2021 to January 29, 2022) (16). We performed an ad-hoc subgroup analysis on children who had a HCW-PCR with Ct <33 and with Ct <30. Data were entered by using an electronic case report form (PHP/MySQL) and were analyzed by using Stata/SE v15 (StataCorp, College Station, TX, USA). Quantitative data were compared by Student *t* test and categorical data by chi-squared or Fisher exact test.

## Results

Among the 836 patients with a SCANS-RAT (median 4.0 years, interquartile range 2-7), 774 (92.6%) were symptomatic. In addition to a SCANS-RAT, 263 children had a HCW-PCR, 589 children a HCW-RAT, and 4 children both tests. Patients characteristics are detailed in Table 1. The prevalence of SARS-CoV-2 infection was 18.4% (154/836) (95%CI 15.8; 21.2) during the whole study period: 10.1% (62/617) (95%CI 7.8; 12.7) in period 1 (Delta wave) and 42.0% (92/219) (95%CI 35.4–48.8) in period 2 (Omicron wave).

**Table 1:** Characteristics of patients included in the study (*N* = 836).

**Test performed**	**HCW-RAT**	**HCW-PCR**	**SCANS-RAT**
	***N =* 593**	***N =* 267**	***N =* 836**
Age in years, median (IQR)	4 (2–7)	4 (2–8)	4 (2–7)
Sexe (male), *n* (%)	321 (54.1%)	148 (55.4%)	460 (55.0%)
Positive test, *n* (%)	80 (13.5%)	81 (30.3%)	131 (15.7%)
Contact with confirmed COVID-19 positive case and asymptomatic, *n* (%)	3 (9.1%)	3 (12.5%)	6 (10.9%)
Contact with confirmed COVID-19 positive case and symptomatic, *n* (%)	42 (40.4%)	49 (52.1%)	76 (40.0%)
No contact with confirmed COVID-19 positive case and symptomatic, *n* (%)	34 (7.5%)	28 (19.3%)	48 (8.2%)

4 children had both test, data on contact with confirmed COVID-19 case was missing for 7 children.

**Figure 1:**
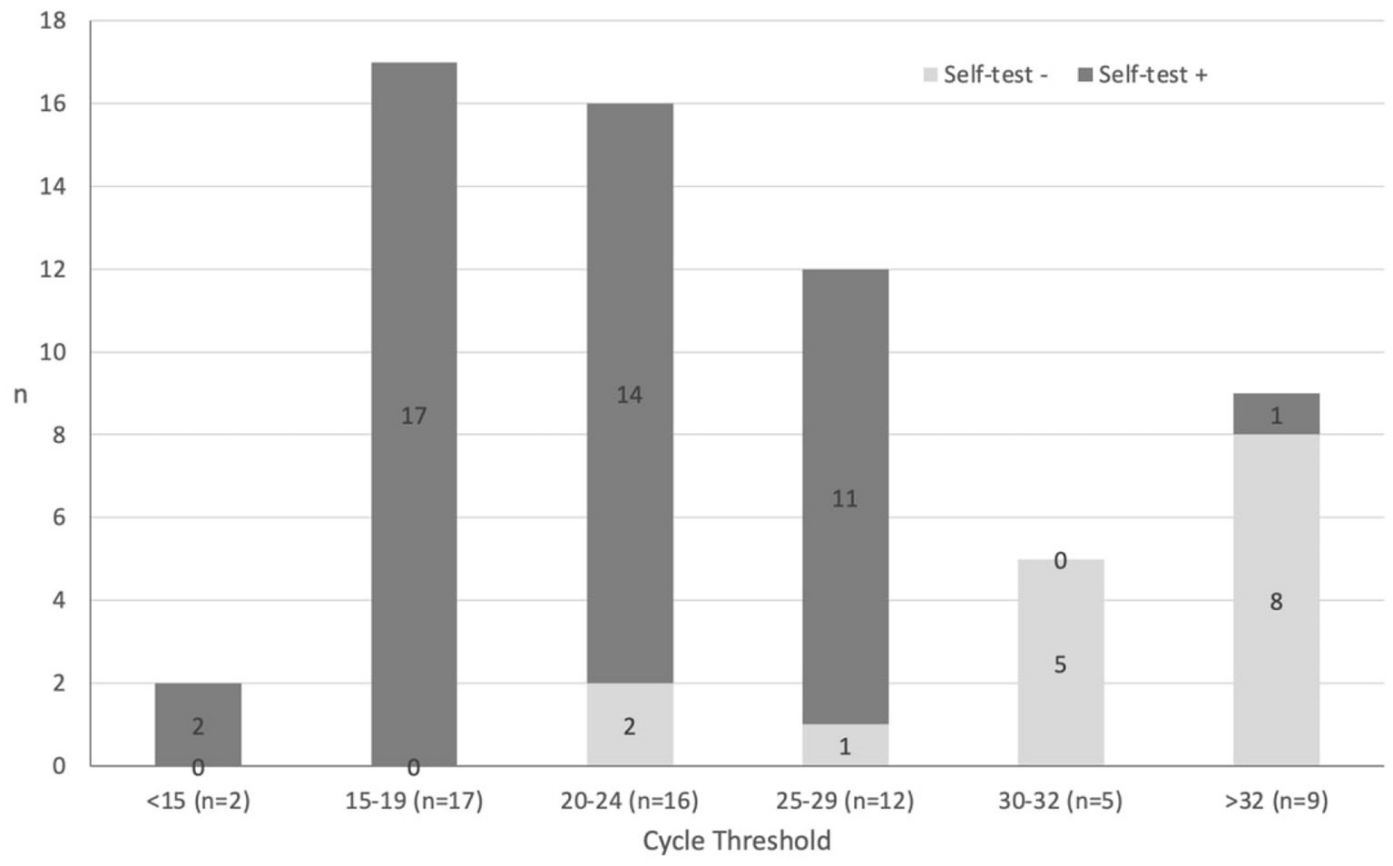
Rapid antigen test from self-collected anterior nasal swabs (self-test) results according to HCW-PCR viral load.

The cycle threshold (Ct) was available for 75.3% (61/81) of positive HCW-PCR. The Table 2 shows the performance of the SCANS-RAT compared to HCW-PCR and HCW-RAT. The overall sensitivity of the SCANS-RAT compared to any positive HCW-PCR results was 70.4% (95%CI 59.2; 80.0). False negative SCANS-RAT results compared to HCW-PCR (9.0%, *n* = 24/267) corresponded mainly to HCW-PCR tests with Ct ≥ 30 (Figure 1). Thus, sensitivity was 84.6% (95%CI 71.9; 93.1) and 93.6% (95%CI 82.5; 98.7) when considering only HCW-PCR with Ct <33 and with Ct <30, respectively. The specificity was always high (from 97.4 to 97.8%). The median delay between the SCANS-RAT and the HCW-PCR was 0 day (interquartile range 0–0). This delay was similar between positive and negative HCW-PCR (*p* = 0.50).

**Table 2:** Performance of the rapid antigen test from self-collected anterior nasal swabs (SCANS-RAT) test compared to reverse transcription polymerase chain reaction from nasopharyngeal swabs (HCW-PCR), and rapid antigen test from nasopharyngeal swabs collected by healthcare workers (HCW-RAT) (*N* = 836).

**SCANS-RAT test compared to**	**Sensitivity**	**Specificity**	**PPV**	**NPV**	**LR+**	**LR-**	**Accuracy**
HCW-RAT (80/593)	91.3 (82.8; 96.4)	99.6 (98.6; 100)	97.3 (90.7; 99.7)	98.6 (97.2; 99.5)	234.0 (58.6; 935)	0.09 (0.04; 0.18)	98.5 (97.1; 99.3)
All HCW-PCR (81/267)	70.4 (59.2; 80.0)	97.8 (94.6; 99.4)	93.4 (84.1; 98.2)	88.3 (83.2; 92.4)	32.7 (12.3; 87.2)	0.30 (0.22; 0.42)	89.5 (85.2; 92.9)
HCW-PCR with Ct <33 (52/267)	84.6 (71.9; 93.1)	97.4 (94.1; 99.2)	89.8 (77.8; 96.6)	96.0 (92.2; 98.2)	33.0 (13.8; 79.0)	0.16 (0.08; 0.30)	94.7 (91.2; 97.2)
HCW-PCR with Ct <30 (47/267)	93.6 (82.5; 98.7)	97.5 (94.3; 99.2)	89.8 (77.8; 96.6)	98.5 (95.6; 99.7)	37.4 (15.7; 89.3)	0.07 (0.02; 0.20)	96.8 (93.7; 98.6)

Data are values (95% confidence interval).

Ct, cycle threshold; PPV, positive predictive value; NPV, negative predictive value; LR+, positive likelihood ratio; LR-, negative likelihood ratio.

The sensitivity of the SCANS-RAT compared to HCW-RAT results was 91.3% (95%CI 82.8; 96.4) while specificity was 99.6% (95%CI 98.6; 100). Of note, sensitivity was comparable between period 1 (85.2%, 95%CI 66.3; 95.8) and period 2 (94.3%, 95%CI 84.3; 98.8). False negative SCANS-RAT results compared to the HCW-RAT accounted for 7/593 cases.

For children from ≥2 to <6 years old (*n* = 308), the SCANS-RAT was obtained by the children themselves alone in 47.4% (146/308) of cases, while for 52.6% (162/308) of cases, an adult, mostly the accompanying adult, had to help. From age ≥6 years, the self-collecting swab was easily performed by the children themselves in 90.9% (179/197) of cases. On appreciation rating (from 1, very pleasant, to 10, very unpleasant), 77.9% of children chose a score ≤3.

## Discussion

To our knowledge, this is the first large prospective study assessing in real-life the diagnostic accuracy of an SCANS-RAT in a pediatric population. Studies assessing SCANS-RAT diagnosis accuracy have involved only adults (9–12). As compared with the HCW-RAT, for which a positive test result is often associated with live viral culture (17), the diagnostic accuracy of the SCANS-RAT is similar in both sensitivity and specificity. If we consider all positive tests independent of Ct number, as compared with HCW-PCR, the SCANS-RAT had excellent specificity but relatively moderate sensitivity under the minimum performance requirements as recommended by the World Health Organization (6). However, HCW-PCR have been reported to remain positive up to 5 weeks after infection while live virus is usually isolable only during the first week (5). Thus, HCW-PCR without Ct may not be the gold-standard to detect contagious patients. There is a continuous relation between Ct and viral culture with a 33% reduction of the odds of live viral culture for 1-unit increase in Ct (18). Several thresholds have been proposed (18). In France, tests with Ct ≤33 are reported as “positive” whereas tests with Ct > 33 are reported as “weak positive” (19). In our study, if we consider only patients with Ct <33, which suggests a high viral load, the sensitivity was good [84.6% (95%CI 71.9; 93.1)]. Viral load is an important determinant of disease transmission, which is a critical parameter for implementing control measures and disease modeling (20, 21). The purpose of the SCANS-RAT is more to detect the most infectious patients than to accurately diagnose COVID-19.

Rapid antigen tests have multiple advantages: suitability, speed of the results and cost. Furthermore, tests from anterior nasal swabs are suitable for repeated tests in children. Indeed, during the successive epidemic waves, children had to undergo many tests, sometimes for a short period of few weeks, and good acceptability is a crucial goal: lower sensitivity of individual tests can be compensated for by frequency of testing and wider dissemination of tests. Because children show substantially reduced mortality from COVID-19, entry screening into schools might require greater compromise that balances resources and sensitivity to testing as many individuals as possible. Because of a high specificity, the risk of a false positive test due to repeated SCANS-RAT is low. The use of tests from self-collected anterior nasal swabs and not from nasopharyngeal swabs collected by healthcare workers is the first step to succeed in a large-scale testing strategy allowing for widespread school opening. Repeated use of the SCANS-RAT can contribute to a wider opening of schools with expected benefits for the mental and physical health of children (22). In the United States, many schools offered free COVID-19 tests (23). In France, in early January 2022, with the Omicron wave, the testing strategy for children at school was difficult to perform: 3 tests in 5 days (24). Indeed, in this context, even if it means losing slightly sensitivity, it appears crucial to have a very good acceptance of the tests in children, allowing a wide use within families without healthcare workers support. Of note, the sensitivity of the SCANS-RAT compared to HCW-PCR and HCW-RAT did not change significantly during the delta and Omicron periods. Similar results were recently reported in a study mainly in adult population (25).

Our study has some limitations. First, we did not use centralized reverse transcription polymerase chain reaction performed by centralized high-complexity laboratories and the Ct number was available for only three-quarters of SARS-CoV-2–positive patients. However, this limitation is also a strength of our real-life study: we compared the SCANS-RAT with the methods used in real life. Second, most children in our study were symptomatic (92.7%), and we cannot extrapolate our results for screening in asymptomatic children. However, the accuracy of the HCW-RAT and contagiousness are believed to be mainly driven by the viral load and SCANS-RAT used in our study has as good sensitivity as HCW-PCR with low Ct (5). Third, for some children, HCW-RAT and HCW-PCR were not performed the same day. However, the majority of HCW-PCR were performed in symptomatic children and in the first few days after the symptom onset. This corresponds to a period where children have high viral loads with Ct <30 (26).

In conclusion, the anterior nasal self-collected test used in this study seems reliable and suitable, allowing to detect infectious children. A repeated screening strategy using SCANS-RAT can play a major role in controlling the pandemic.

## Data availability statement

The data that support the findings of this study are available from the corresponding author upon reasonable request.

## Ethics statement

The study protocol was approved by an Ethics Committee (Centre Hospitalier Intercommunal de Créteil, France) and was registered at ClinicalTrials.gov: NCT0441231. Legal guardians and children were informed with a written non-opposition form. Written informed consent was not required to participate in this study in accordance with the national legislation and the institutional requirements.

## Author contributions

RC, SB, CJ, and CL designed the study. CA, AF, FC-S, OR, AA, CB, and BV made the acquisition of the study data. RC, AR, SB, and CL drafted the initial manuscript and agree to be accountable for all aspects of the work in ensuring that questions related to the accuracy or integrity of any part of the work are appropriately investigated and resolved. All the authors analyzed the data, revised critically the manuscript for important intellectual content, and provide approval for publication of the content. All authors contributed to the article and approved the submitted version.

## Funding

This study has been self-funded by ACTIV, including the purchase of the tests.

## Conflict of interest

The authors declare that the research was conducted in the absence of any commercial or financial relationships that could be construed as a potential conflict of interest.

## Publisher's note

All claims expressed in this article are solely those of the authors and do not necessarily represent those of their affiliated organizations, or those of the publisher, the editors and the reviewers. Any product that may be evaluated in this article, or claim that may be made by its manufacturer, is not guaranteed or endorsed by the publisher.

## Abbreviations

Ct: cycle threshold
HCW-RAT: rapid antigen test from nasopharyngeal swabs collected by healthcare workers
HCW-PCR: reverse transcription polymerase chain reaction from nasopharyngeal swabs collected by healthcare workers
SCANS-RAT: rapid antigen test from self-collected anterior nasal swabs.
